# Ecological isolation by the Chishui River drives microecology divergence in Daqu

**DOI:** 10.3389/fmicb.2026.1786069

**Published:** 2026-03-12

**Authors:** Dandan Song, Xian Zhong, Yaling Mu, He Huang, Liang Yang

**Affiliations:** 1 School of Brewing Engineering, Moutai Institute, Renhuai, China; 2 Guizhou Key Laboratory of Microbial Resources Exploration in Fermentation Industry, Kweichow Moutai Group, Zunyi, China; 3 Kweichow Moutai Co. Ltd., Renhuai, China; 4 Department of Food Science and Technology, School of Agriculture and Biology, Shanghai Jiao Tong University, Shanghai, China

**Keywords:** ecological isolation, high-temperature Daqu, microecology, multivariate statistical analysis, Qu-omics

## Abstract

This study investigates how the Chishui River acts as a geographical barrier driving microecological divergence in Daqu. Utilizing qu-omics, this study investigated metabolic phenotypes, microbial community composition, and functional differentiation in Daqu from both riverbanks. Results showed significant geographical variations were found in volatile profiles and dominant genera (e.g., *Kroppenstedtia, Aspergillus*). Network analysis revealed that positive bacterial interactions increased from the right to the left bank, while fungal cooperation markedly decreased. Right-bank Daqu exhibited upregulated oxidative phosphorylation, starch, sucrose, and glutathione metabolism, driven by stochastic fungal assembly. Conversely, left-bank Daqu was characterized by enhanced purine, monobactam, and nitrogen metabolism under deterministic assembly. This study introduces the concept of “ecological isolation” to Daqu microecology for the first time. It highlights how geographical barriers drive functional differentiation, offering vital implications for the regional regulation of Baijiu quality.

## 1 Introduction

Baijiu is a traditional Chinese alcoholic beverage with a history of over 2000 years, produced through fermentation, distillation, aging, and blending (Miao et al., 2022; Xu et al., 2022). Twelve distinct baijiu flavor types are recognized based on their production processes and sensory characteristics. Among them, sauce-aroma baijiu is especially popular with consumers for its prominent sauce-like aroma and persistent aftertaste (He et al., 2025; Yang et al., 2025). As one of the four fundamental flavor types of Baijiu in China, sauce-flavor Baijiu contributes approximately 40% of the industry's profits and has become a major economic pillar for Guizhou Province (Mu et al., 2025a). The complex flavor profile of sauce-flavor Baijiu originates from its unique manufacturing process, in which Daqu serves as both a saccharification and fermentation agent. Specifically, Daqu provides essential enzymes, microbiota, and flavor compounds during fermentation (Huang et al., 2024; Mu et al., 2025b). Consequently, the quality of Daqu substantially determines the final quality of Baijiu.

Numerous efforts have accumulated evidences regarding the factors influence Daqu micro-ecology including its microbiota composition, volatile compounds, and metabolome profiles. These factors encompass environmental conditions (temperature, humidity; Yang et al., 2024a), seasonal variations (Yang S. et al., 2024; Yang et al., 2024b), production processes (bulk density, storage conditions, and raw materials; Wang et al., 2018), and geographical origins (Zhu et al., 2023).

Among these, geographical divergence can affect both the metabolome and microbiota of Daqu. For example, although both being classified as high-temperature sauce-flavor Daqu, samples from Guizhou contain higher concentrations of pyrazines (specifically 2,6-dimethylpyrazine, 2,3,5-trimethylpyrazine, and tetramethylpyrazine) than those from Sichuan (Fangjian et al., 2023). Similarly, geographic divergence exists even within the same province; for example, Renhuai high-temperature Daqu (HTD) is characterized by bacterial groups like *Oceanobacillus* and *Bacillus*, while Jinsha HTD is dominated by *Thermoactinomyces* and *Weissella*. These localized variations suggest that subtle environmental or physical barriers may drive microecological differentiation even within a single basin (Tang et al., 2025).

The Chishui River Basin is a renowned production area for sauce-flavor Baijiu and its essential starter culture, high-temperature Daqu (HTD). Geographically, the basin is characterized by a unique “two mountains sandwiching one river” topography, forming a deep, enclosed canyon. This distinctive landscape creates a stable and specialized microclimate, characterized by high temperatures, high humidity, and stagnant air, which serves as a “natural incubator” for the enrichment and long-term survival of the heat-tolerant microbiota essential for high-temperature Daqu production. Furthermore, this canyon environment provides the physical foundation for the dispersal limitation explored in this study, where the river functions as a primary biogeographical barrier that restricts microbial movement and shapes the distinct regional microecological fingerprints observed on opposite banks.

In natural ecosystems, “reproductive isolation” describes an isolating mechanism where closely related species cannot mate successfully, produce viable offspring, or generate infertile hybrids due to biological barriers (Jarrett et al., 2025). This phenomenon has been extensively documented in various species including insects and fish in natural environments (Aguillon et al., 2025; Hsu et al., 2024). Building on this concept, we define “ecological isolation” as divergence in microbial ecology between adjacent Daqu production habitats driven by geographic barriers (e.g., rivers or mountains). Geographical origin is well-recognized as a primary factor influencing Daqu quality. However, most existing studies have compared Daqu from vast distances, where differences in climate and raw materials often mask the subtle effects of physical barriers. A significant research gap exists regarding the impact of microgeographical isolation within a single river basin on microbial assembly. The Chishui River Basin provides an ideal setting to investigate this. Although distilleries on opposite banks share identical climatic conditions and traditional techniques, the river may act as a primary biogeographical barrier that restricts microbial dispersal.

Advanced omics technologies have become essential for dissecting the complex microbial and metabolic networks in high-temperature Daqu (HTD; Ferrocino et al., 2023). Through an integrated multi-omics framework known as Qu-omics (Ferrocino et al., 2023; Yang et al., 2024a), researchers can simultaneously characterize microbial composition and function, metabolic phenotype, thereby enabling a comprehensive evaluation of microecological heterogeneity. Recent advances in flavor analysis technologies, particularly gas chromatography-mass spectrometry (GC-MS), have enhanced the characterization of Daqu's flavor profiles (Yang et al., 2022; Yang S. et al., 2024, 2023). Concurrently, liquid chromatography-mass spectrometry (LC-MS)-based metabolomics has advanced our understanding of Daqu's metabolic profiles, revealing key mechanisms underlying flavor formation (Yang et al., 2021). Metagenomics and metaproteomics provide high resolution taxonomic and functional readouts, identifying taxa and pathways that contribute to flavor development (Yang et al., 2020; Yang L. et al., 2023). Integrating these Qu-omics techniques is therefore crucial for unraveling the metabolic heterogeneity and formation mechanisms of Daqu produced on the two banks of the Chishui River.

**Figure 1:**
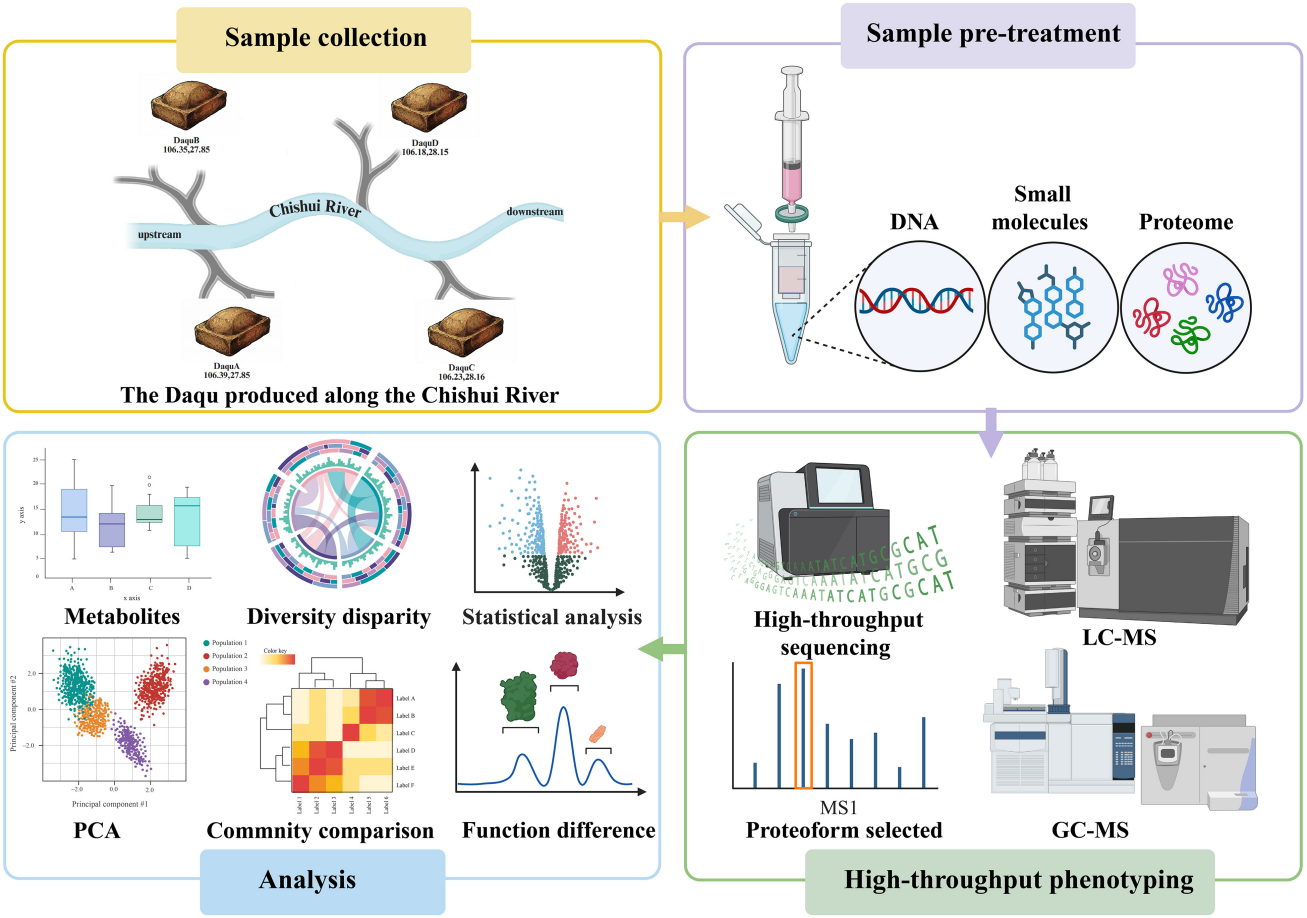
Workflow of this study.

In this study, we employed an integrated multi-omics approach combining metagenomics, volatile metabolomics, non-targeted metabolomics, and metaproteomics to comprehensively analyze the microbial community structure, metabolic composition, and expressed enzyme repertoire of Daqu produced on both sides of the Chishui River (Figure 1). Bioinformatics analyses and ecological modeling were applied to elucidate the microecological differentiation and underlying formation mechanisms resulting from the “ecological isolation” effect created by this natural geographical barrier. Our findings provide novel insights into the formation mechanisms and functional differentiation of microbial communities in Daqu, while offering valuable guidance for optimizing Daqu's brewing functionality through improved quality control practices.

## 2 Materials and methods

### 2.1 Daqu samples

Daqu samples were collected from four distilleries located along the Chishui River in northern Guizhou Province, China. The sampling sites represented distinct geographical positions relative to the river: two distilleries on the right bank (Daqu_A, upstream; Daqu_C, downstream) and two on the left bank (Daqu_B, upstream; Daqu_D, downstream). To ensure consistency across all experimental groups, all samples were collected in March following a standardized production cycle: 40 days of high-temperature fermentation followed by a 6-month maturation (storage) period. All sampling was conducted under similar environmental conditions.

Each distillery provided nine Daqu subsamples obtained from nine independent fermentation rooms. Every three subsamples were pooled to generate one composite sample, resulting in three composite samples per distillery, and 12 composite samples in total. All subsamples were crushed and homogenized, and approximately 500 g of powder was obtained from each subsample using the quartering method. Each composite sample was divided into two aliquots for metabolomics analysis (stored at 4°C) and microbiome analysis (stored at −80°C), respectively.

### 2.2 Volatilome analysis using GC-MS

Volatile compounds in Daqu samples were analyzed via headspace solid-phase microextraction (HS-SPME) coupled with gas chromatography-mass spectrometry (GC-MS). Approximately 2.0 g of sample was weighed into a 20 mL headspace vial, and mixed with 10 μL of internal standard solution (4-octanol, 20 μg/mL). Volatiles were extracted using a DVB/CAR/PDMS SPME fiber (50/30 μm × 2 cm; Supelco) on a GERSTEL Multi-Purpose Sampler (MPS2).

Samples were equilibrated at 40°C for 20 min and extracted at the same temperature for 40 min. Following extraction, the SPME fiber was desorbed in the GC injector at 250°C for 5 min. Volatile compounds were separated and detected using an Agilent 7890B-5977A GC-MS system equipped with an Agilent DB-WAX column (60 m × 0.25 mm × 0.25 μm; Yang et al., 2022). High-purity helium (>99.99%) was used as the carrier gas at a constant flow rate of 1.0 mL/min.

The GC oven temperature program was as follows: initial hold at 40°C for 2 min, ramping to 150°C at 2°C/min, then to 230°C at 5°C/min, followed by a final hold at 230°C for 8 min. The transfer line temperature was maintained at 250°C, and the MS ion source temperature was set at 230°C. Electron-impact (EI) ionization was performed at 70 eV, and mass spectra were acquired across m/z 35–450 in splitless injection mode. All analyses were performed in triplicate. Volatile compounds were identified by comparing mass spectra with the NIST 2020 database, and quantification was conducted using the internal standard method (Yang et al., 2022).

### 2.3 Metagenome sequencing and data analysis

Genomic DNA was extracted using the Omega Soil DNA Kit (Omega Bio-tek, Norcross, USA) following the manufacturer's instructions. DNA quality and concentration were assessed by 1% (w/v) agarose gel electrophoresis and a NanoDrop 2000 spectrophotometer (Thermo Scientific, USA).

Qualified DNA samples were fragmented to an average size of 350 bp using a Covaris M220 system (Gene Company Limited, China) for paired-end library construction. Libraries were prepared using the NEXTFLEX Rapid DNA-Seq Kit (Bioo Scientific, USA) and sequenced on an Illumina NovaSeq X Plus platform (Illumina Inc., USA) with 150 bp paired-end reads. The target sequencing depth for each of the 12 composite samples was approximately 12 Gb, generating a total dataset of 144 Gb.

Raw sequencing data were processed using Trimmomatic (v0.36) to obtain high-quality clean reads. Quality-filtered data were assembled *de novo* using MEGAHIT (v1.1.2). Open reading frames (ORFs) were predicted from assembled contigs using Prodigal (v2.6.3) with a minimum length threshold of 100 bp. A non-redundant gene catalog was constructed using CD-HIT (v4.7) with 90% sequence identity and 90% coverage thresholds. Gene abundance was estimated using SOAPaligner (v2.21) with 95% identity criteria. The resulting non-redundant gene set was used for subsequent proteomic analysis. Sequencing and initial bioinformatics analyses were performed by Shanghai Bean Biotechnology Co., Ltd.

### 2.4 Metaproteomic assay and data analysis

Protein extraction from Daqu samples was performed using the Tris-phenol method. Protein quality was verified by sodium dodecyl sulfate–polyacrylamide gel electrophoresis (SDS-PAGE). Qualified proteins were subsequently reduced, alkylated, and digested with trypsin, followed by desalting using Waters Oasis HLB solid-phase extraction columns.

Desalted peptides were quantified and analyzed by liquid chromatography-tandem mass spectrometry (LC-MS/MS) using an Easy-nLC 1,200 nanoflow LC system coupled to a Q-Exactive mass spectrometer (Thermo Fisher Scientific, USA). Mass spectrometry data were processed against a custom protein database derived from Daqu metagenomic sequences. All protein detection and analytical procedures followed established protocols from our laboratory (Yang et al., 2024a).

### 2.5 Metabolomics analysis based on LC-MS/MS

Non-volatile metabolites were extracted and analyzed according to a previously established method with slight modifications (Yang et al., 2021). Briefly, a mixture of deuterated internal standards consisting of hexanoic acid-d_11_, benzoic acid-d_5_, cholic acid-d_4_, 2-amino-3-(2-chlorophenyl)-propanoic acid, D-luciferin, testosterone-d_3_, and L-tryptophan-d_5_ (final concentration: 0.2 mg/L each) was prepared. Daqu samples (100 ± 1 mg) were mixed with beads and 500 μL of extraction solution (methanol: water = 7:3, v/v) containing the internal standards.

After vortexing for 3 min, samples were sonicated in an ice bath for 10 min, vortexed for an additional 1 min, and then incubated at −20°C for 30 min. The samples were centrifuged at 12,000 rpm for 10 min at 4°C. The supernatant was collected and recentrifuged at 12,000 rpm for 3 min at 4°C to remove residual particulates. A 200 μL aliquot of the final supernatant was transferred for UPLC-MS analysis.

Chromatographic separation was performed on a Waters ACQUITY Premier HSS T3 column (1.8 μm, 2.1 × 100 mm) maintained at 40°C. For positive ion mode analysis, the mobile phase consisted of 0.1% (v/v) formic acid in water (solvent A) and 0.1% (v/v) formic acid in acetonitrile (solvent B). The gradient elution program was as follows: 5%−20% B (0–2 min), 20%−60% B (2–5 min), 60%−99% B (5–6 min), 99% B (6–7.5 min), followed by re-equilibration to 5% B (7.5–7.6 min) and a 2.4-min hold. The flow rate was 0.4 mL/min with an injection volume of 4 μL. Negative ion mode analysis employed an identical gradient profile.

Mass spectrometric detection was conducted in both positive and negative ESI modes with alternating full-scan MS and data-dependent MS acquisition. Full-scan spectra were acquired at a resolution of 35,000 over m/z 75–1000. Key parameters included: spray voltage 3.5 kV (positive) or 3.2 kV (negative); sheath gas 30 Arb; auxiliary gas 5 Arb; ion transfer tube temperature 320°C; vaporizer temperature 300°C. Data-dependent scans employed dynamic exclusion (3 s) with collision energies of 30, 40, and 50 eV, triggered at a threshold of 1.0 × 106 cps. The top 10 most intense ions were selected for fragmentation. Raw data were processed using Mass Hunter Profinder (v10.0.2) for automated metabolite feature extraction, including peak alignment, peak picking, feature matching, and semi-quantitative analysis (Yang et al., 2021). Metabolite identification was performed by matching MS/MS spectra against the METLIN metabolite database and an in-house library. The identified metabolites were annotated according to Metabolomics Standards Initiative (MSI) levels: Level 1 identification (high confidence) was based on accurate mass measurements and MS/MS fragmentation spectra matching with available standards; Level 2 annotation (moderate confidence) was based on accurate mass and comparisons with spectral libraries without pure standards for verification.

### 2.6 Statistical analysis

Principal component analysis (PCA), partial least squares-discriminant analysis (PLS-DA), and permutation tests were performed using the online analysis platform OmicStudio (https://www.omicstudio.cn/tool). Pathway analysis and enrichment analysis of differentially expressed metabolites were conducted using MetaboAnalyst 5.0 (https://www.metaboanalyst.ca/). To quantitatively assess the relative importance of deterministic and stochastic processes in community assembly, the Normalized Stochasticity Ratio (NST) analysis was employed based on a general null model framework. This method effectively normalizes ecological stochasticity to a range of 0%−100%, providing a comparable metric across microbial communities. Following established standards, a 50% boundary was utilized as the critical point: text {NST} < 50% indicates that deterministic processes (such as environmental filtering or biotic interactions) primarily govern the assembly, whereas text {NST} >50% indicates that stochastic processes (such as dispersal limitation or ecological drift) are the dominant drivers.

## 3 Results and discussion

### 3.1 Comparison of volatile compound composition

Gas chromatography-mass spectrometry (GC-MS), a widely used technique for flavor characterization, was employed to profile volatile compounds in Daqu (Xiao et al., 2025). A total of 158 volatile compounds were detected, including 18 alcohols, 4 acids, 25 esters, 33 aldehydes and ketones, and 15 pyrazines. These results were consistent with previous reports on regional Daqu (Li et al., 2024; Tang et al., 2025), indicating that dominant volatile profiles are largely conserved across production regions.

**Figure 2:**
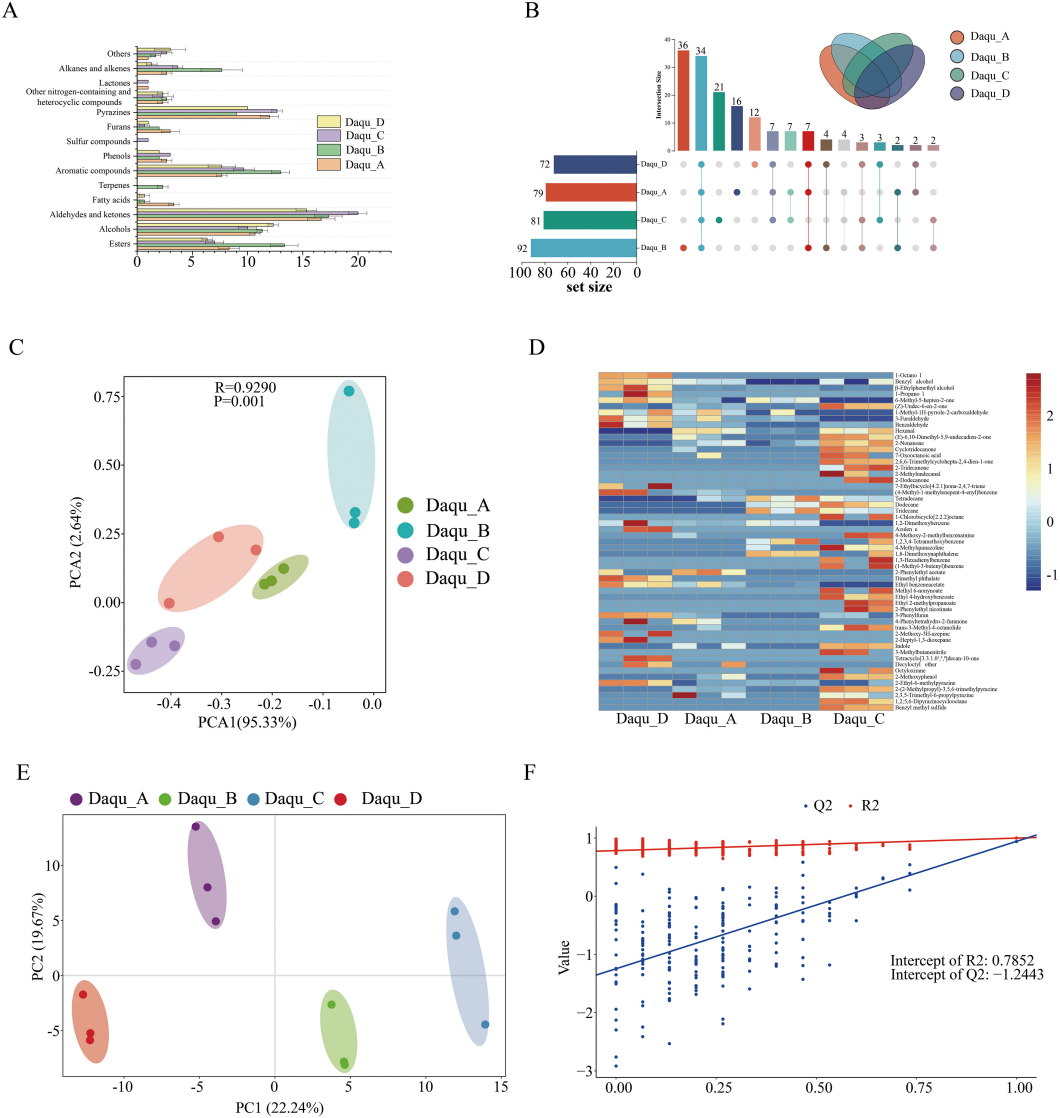
Composition of compounds in soy sauce aroma and flavor type Daqu. **(A)** The number of different classes metabolites in Daqu. **(B)** Upset plot of Daqu compounds. **(C)** PCA plot of volatiles in Daqu. **(D)** Heatmap visualization of the significantly differential volatile compounds identified with a Variable Importance in Projection (VIP) score > 1.0. The color scale (from blue to red) represents the relative abundance of metabolites, standardized by Z-scores. **(E)** Partial Least Squares-Discriminant Analysis (PLS-DA) score plot illustrating the distinct clustering and robust separation of the four Daqu groups based on their volatilome profiles. **(F)** Permutation test (*n* = 200) used to validate the PLS-DA model. The R^∧^2 and Q^∧^2 values indicate the goodness-of-fit and the predictability of the model, respectively, with the intercept values confirming that the model is not overfitted. The horizontal bars on the left represent the total number of compounds identified in each sample set. The vertical bars at the top indicate the number of compounds in each specific intersection, with the filled black circles in the matrix below representing the groups included in that intersection. The “core” compounds shared by all samples are shown in the intersection containing all groups, while unique compounds specific to a single group are indicated by single-point intersections.

Esters, aldehydes, and ketones represented the most abundant compound classes across all four Daqu samples. While Daqu_A and Daqu_C showed comparable volatile diversity, Daqu_B exhibited the greatest number of compounds. This variation may be attributed to geographical factors: Daqu_A and Daqu_C originate from distilleries on the same riverbank, whereas Daqu_B was produced on the opposite side. The river likely functions as an ecological barrier, contributing to the distinct microbial, and metabolic diversity observed in Daqu_B. In contrast, Daqu_D displayed fewer volatile compounds despite sharing the same riverbank with Daqu_B, a phenomenon requiring further investigation (Figures 2A, B).

Although Daqu_B showed the highest volatile diversity, the total volatile content was higher in Daqu_C and Daqu_D (Supplementary Table 1). Notably, Daqu_A and Daqu_C displayed similar total volatile profiles, suggesting that geographical isolation may influence compound diversity more than overall abundance.

A core set of 34 volatile compounds was common to all Daqu samples, representing the conserved volatilome of high-temperature Daqu in this region. This core group includes key aroma contributors such as β-ethylphenethyl alcohol, 3-methyl-1-butanol, 3-methylbutanal, benzeneacetaldehyde, ethyl hexadecanoate, and phenol (Figure 2B). These compounds, derived from essential microbial pathways such as amino acid degradation and fatty acid metabolism, provide the fundamental sensory framework for sauce-flavor Baijiu, regardless of the geographical variations between riverbanks. Several unique compounds were exclusively detected in specific samples: ethyl nonanoate and ethyl (E)-4-decenoate in Daqu_B, and 2-ethenyl-6-methylpyrazine and ethyl 4-hydroxybenzoate in Daqu_C.

Principal component analysis (PCA) revealed substantial differences in volatile profiles among the four Daqu samples. The first two principal components (PC1 and PC2) accounted for 95.33 and 2.64% of the total variance, respectively (Figure 2C), indicating significant compositional distinctions.

As shown in Figures 2D–F, partial least squares-discriminant analysis (PLS-DA) identified 55 significantly differential compounds among the four Daqu samples, including aldehydes and ketones (14), aromatic compounds (8), esters (7), and alcohols (4). Notably, nearly all alcohols were more abundant in Daqu_D, while pyrazines predominated in Daqu_A and Daqu_C. Additionally, certain alkanes and alkenes were more prevalent in Daqu_B (Figure 2D). Among differential compounds, ethyl esters, which impart fruity or floral aromas and are primarily produced by yeasts, were particularly abundant in Daqu_C (Mu et al., 2023; Xu et al., 2022).

Specific flavor compounds showed distinct distribution patterns: benzyl alcohol (1.17 ± 0.15 μg/g), derived from phenylalanine and contributing floral and balsamic notes, was mainly enriched in Daqu_D. Conversely, hexanal (0.62–0.64 μg/g), produced from linolenic acid and imparting grassy odors, was more abundant in Daqu_A and Daqu_C. These compounds have been recognized as important flavor contributors in Daqu (Ma et al., 2022; Zhai et al., 2022).

The “two mountains sandwiching one river” topography likely drives this bank-level divergence by creating a confined canyon that restricts regional airflow and microbial dispersal. These physical and microclimatic barriers (e.g., humidity and temperature) impose significant dispersal limitations, allowing the river to function as a primary ecological filter that shapes the distinct microbial successions and metabolic profiles observed on opposite banks. Collectively, these findings demonstrate that geographical location may influences the microecological marker profiles of Daqu. All identified markers represent key metabolites in sauce-aroma baijiu fermentation, with most directly contributing to the final liquor's flavor characteristics. Consequently, understanding and controlling geographical effects could provide an effective strategy for regulating the formation of specific microecological markers and ultimately shaping the flavor profile of baijiu.

### 3.2 Analysis of non-volatile metabolites by LC-MS/MS

#### 3.2.1 Differences in metabolite profiles among Daqu samples

Non-volatile metabolites in the four Daqu types were analyzed using LC-MS/MS. A total of 2,811 and 2,272 metabolite ion features were detected in ESI+ and ESI– modes, respectively. After MS/MS spectral matching, 479 metabolites were confidently identified (199 in ESI+ and 280 in ESI–). These comprised 124 organic acids and derivatives, 112 amino acids and derivatives, 69 small peptides, 50 heterocyclic compounds, 36 fatty acids, 28 sugars and sugar derivatives, 26 phenols, 12 aldehydes and ketones, 10 alcohols, 9 esters, and 3 diterpenoids.

Principal Component Analysis (PCA) based on the identified metabolites revealed distinct clustering of Daqu samples in both ionization modes (Supplementary Figures 1A, B). The first two principal components explained 99.19 and 89.77% of the total variance in ESI+ and ESI– modes, respectively, indicating high instrumental stability and data reliability (Mu et al., 2025b). PCA results indicated that non-volatile metabolomes of Daqu_B and Daqu_D were closely clustered, while Daqu_A and Daqu_C formed separate clusters in both ionization modes. The predominant non-volatile compounds in both modes were amino acids and derivatives, small peptides, heterocyclic compounds, organic acids and derivatives, and fatty acids, collectively accounting for over 80% of the total metabolite content across all samples (Supplementary Figures 1C–F). These findings are consistent with previous metabolomic studies of Daqu (Mu et al., 2023; Qin et al., 2024).

Notably, the total concentration of non-volatile metabolites was highest in Daqu_C and lowest in Daqu_A (Supplementary Figure 1F), while Daqu_B and Daqu_D exhibited comparable intermediate levels. To identify compounds contributing to these metabolic differences, we employed partial least squares discriminant analysis (PLS-DA) followed by 200 permutation tests (Supplementary Figures 2A–F). This analysis identified 66 and 85 differential compounds with variable importance in projection (VIP) scores >1.0 from ESI+ and ESI– models, respectively (Supplementary Figures 2E, F).

#### 3.2.2 Identification of differential metabolites and enrichment analysis

**Figure 3:**
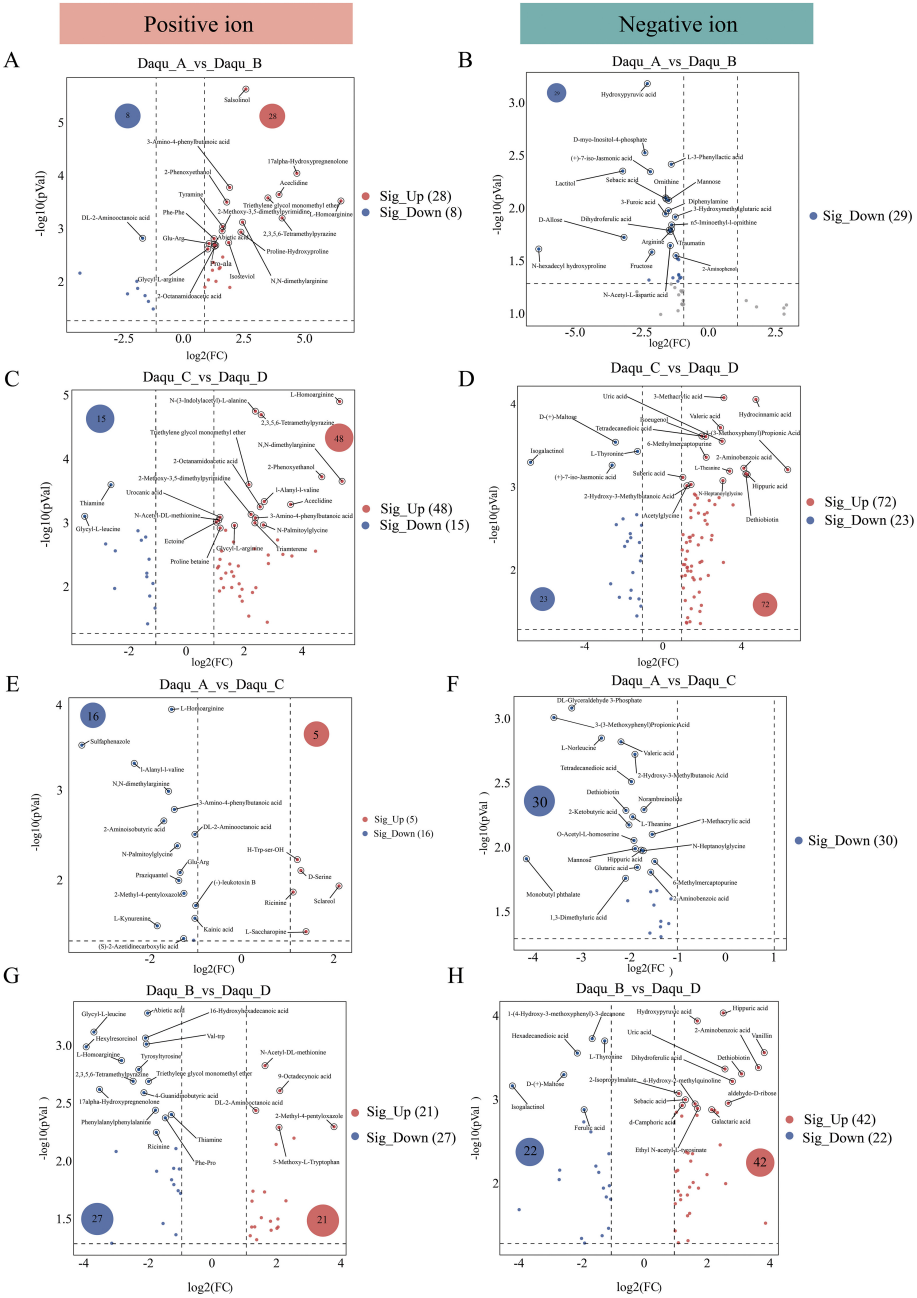
Overview of differences in non-volatile metabolite profiles. **(A–H)** Volcano plot analysis of non-volatile compounds in different Daqu samples, the red point is up-regulated, the blue point is down-regulated.

Volcano plots with thresholds of fold change >2 and *p* < 0.05 were employed to identify differential non-volatile metabolites among Daqu samples (Figures 3A–H). In the positive ion mode, 36, 63, 21, and 48 differential metabolites were identified in the comparisons of Daqu_A vs. Daqu_B, Daqu_C vs. Daqu_D, Daqu_A vs. Daqu_C, and Daqu_B vs. Daqu_D, respectively. Conversely, negative ion mode revealed 29, 95, 30, and 64 differential metabolites in the same respective comparisons. These results demonstrated that sample pairs from the same riverbank exhibited fewer differential metabolites than those from opposite banks, reinforcing the significant impact of river-mediated ecological isolation on Daqu metabolomes.

Venn analysis identified seven significant marker metabolites common to all comparison groups in both ionization modes, including three amino acids/derivatives and four heterocyclic compounds (Supplementary Figures 3A, B). Notably, these compounds were predominantly abundant in Daqu_C but minimal in Daqu_A (Supplementary Figure 3C), explaining the characteristic richness of pyrazines and heterocyclic compounds in Daqu_C.

**Figure 4:**
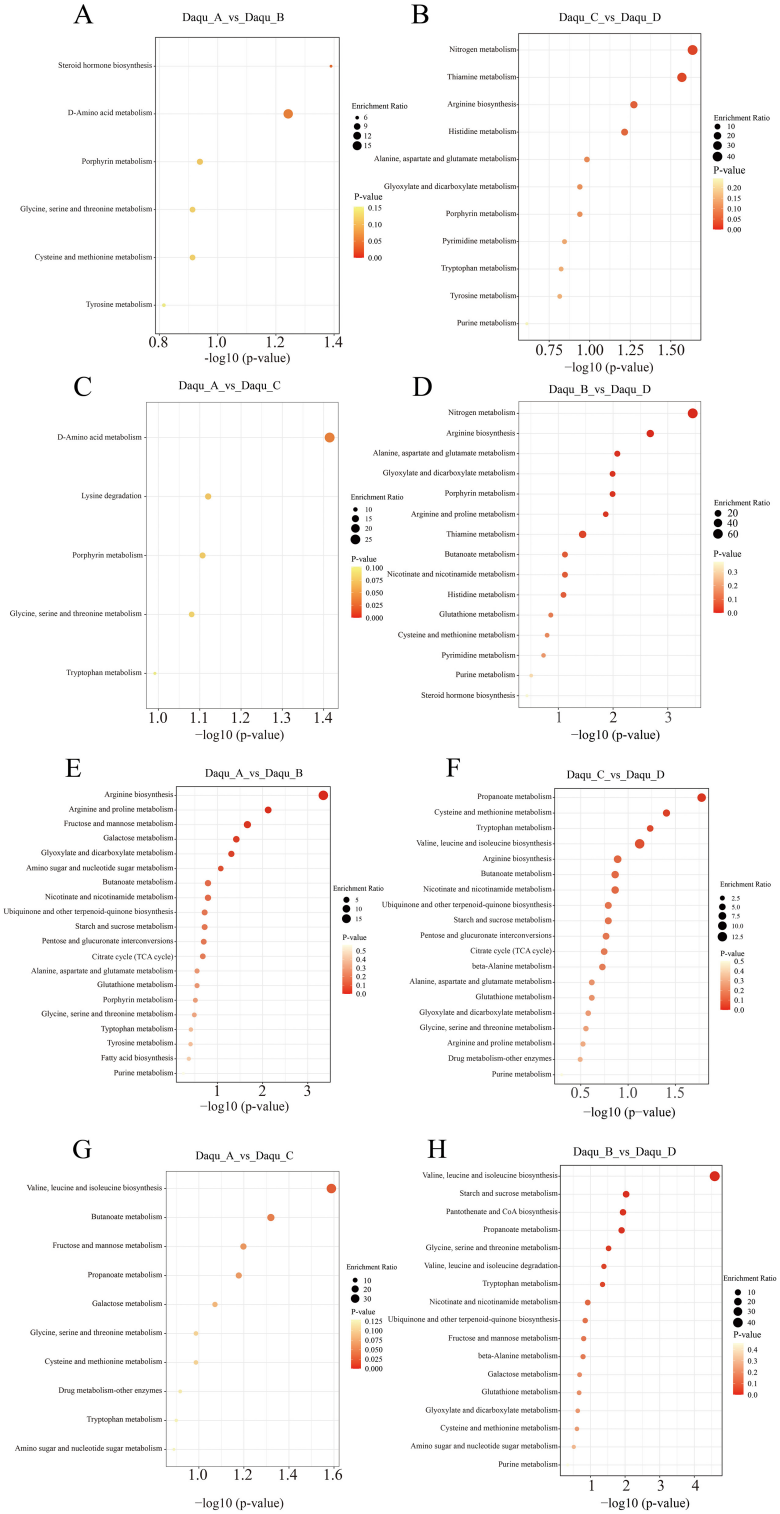
Enrichment analysis of differential metabolites. Positive ion model **(A–D)**, negative ion model **(E–H)**.

Enrichment analysis revealed that non-volatile metabolites were primarily associated with amino acid metabolism (phenylalanine, casein, and tryptophan), glycolysis/TCA cycle, fatty acid metabolism, and phenylalanine/flavonoid/pyrazine secondary metabolism pathways (Figure 4). Distinct enrichment patterns emerged across different Daqu comparisons.

In the Daqu_A_vs._Daqu_B comparison, 26 metabolic pathways showed significant correlations (Figures 4A, E), including arginine biosynthesis, fructose/mannose metabolism, galactose metabolism, and biosynthesis of secondary metabolites. Positive ion mode highlighted divergent flavor precursor synthesis pathways (glycine, serine, threonine, and tyrosine metabolism), while negative ion mode revealed pronounced differences in TCA cycle, arginine/proline metabolism, and carbohydrate utilization pathways. These findings demonstrate fundamental functional divergence between banks in both energy metabolism and flavor compound biosynthesis.

The Daqu_C_vs._Daqu_D comparison showed enrichment of 30 metabolic pathways (11 POS, 19 NEG) (Figures 4B, F). Positive mode analysis indicated significant differences in nitrogen metabolism, amino acid metabolism (histidine, tryptophan, tyrosine, and alanine/aspartate/glutamate), and nucleotide metabolism (purine/pyrimidine). Negative mode analysis revealed substantial variations in central carbon metabolism (TCA cycle, starch/sucrose metabolism, and pentose/glucuronate interconversions) and cofactor/vitamin metabolism (nicotinate/nicotinamide metabolism, and ubiquinone biosynthesis). The significant enrichment of drug metabolism-other enzymes pathway suggested differential xenobiotic biodegradation capacities. This comprehensive metabolic re-programming underscores multifaceted divergence in nitrogen utilization, bioenergetics, biosynthetic processes, and detoxification potential.

For same-bank comparisons, Daqu_A_vs._Daqu_C exhibited enrichment in only 13 metabolic pathways (Figures 4C, G), primarily involving D-amino acid metabolism—indicating bacterial community differences—along with branched-chain amino acid biosynthesis, short-chain fatty acid metabolism, and drug metabolism pathways (Wang et al., 2025). This focused metabolic disparity suggests relatively conserved core functionality between same-bank samples.

Despite geographical proximity, Daqu_B and Daqu_D showed extensive metabolic divergence encompassing nitrogen metabolism, amino acid/nucleotide biosynthesis, central carbon metabolism, and cofactor synthesis (Figures 4D, H). The enrichment of nitrogen metabolism, starch/sucrose metabolism, arginine/proline metabolism, and ubiquinone biosynthesis pathways indicated fundamental differences in nutrient utilization and energy production, suggesting influences beyond geographical location such as micro-environmental variations or stochastic microbial assembly.

Collectively, these results demonstrate that the ecological barrier effect of the Chishui River exerts substantially greater impact on metabolic divergence than intra-bank geographical variation. Opposite-bank comparisons revealed extensive reprogramming of core metabolic pathways including nitrogen assimilation, central carbon metabolism, and cofactor biosynthesis, indicating fundamental differences in microbial functional architecture. In contrast, same-bank comparisons showed fewer metabolic disparities primarily restricted to specialized pathways such as D-amino acid metabolism and flavor precursor synthesis, while maintaining conserved core metabolic networks. These findings establish that river-mediated ecological isolation drives systematic metabolomic differentiation, whereas within-bank variability arises mainly from localized modifications without altering core metabolic functionality.

### 3.3 Metagenomiscs analysis

#### 3.3.1 Overview of sequencing data

Metagenomic sequencing was performed to characterize the microbial community structures of the four Daqu samples (Daqu_A, Daqu_B, Daqu_C, and Daqu_D). As summarized in Supplementary Table 2, the sequencing generated 53.0 Gb of raw data. All samples exhibited high-quality sequencing metrics, with Q20 and Q30 values exceeding 99 and 96%, respectively, indicating excellent data quality suitable for downstream analyses.

After quality control and host DNA filtration, 349,113,490 high-quality clean reads were retained. *De novo* assembly produced contigs for each sample: Daqu_A (107,805 contigs; N50 = 4,476 bp), Daqu_B (195,824 contigs; N50 = 1,921 bp), Daqu_C (122,982 contigs; N50 = 2,096 bp), and Daqu_D (58,521 contigs; N50 = 1,406 bp).

Gene prediction identified 964,343 open reading frames (ORFs) across all samples: Daqu_A (236,244 ORFs), Daqu_B (384,539 ORFs), Daqu_C (252,743 ORFs), and Daqu_D (90,817 ORFs). Subsequent clustering and redundancy removal yielded a non-redundant unigene catalog of 502,748 sequences, with sample-specific contributions of Daqu_A (133,576 unigenes), Daqu_B (185,667 unigenes), Daqu_C (131,956 unigenes), and Daqu_D (51,549 unigenes). This comprehensive metagenomic dataset provides a solid foundation for investigating the functional potential and taxonomic composition of these traditional fermentation starters (Han et al., 2024; Yang et al., 2024a).

#### 3.3.2 Differences in microbial structure within Daqu among four Daqu

Traditional solid-state fermentation environments exhibit pronounced regional characteristics (Han et al., 2024). In this study, a total of 1,196 bacterial genera belonging to 116 classes and 792 fungal genera distributed across 57 classes were identified.

Community richness, as measured by Ace/Chao indices, showed substantial variation. Daqu_C and Daqu_A exhibited similar richness levels, while Daqu_B and Daqu_D displayed significant fluctuations in both bacterial and fungal communities (Supplementary Figure 4). Shannon/Simpson diversity indices revealed that bacterial rare species diversity was lower in Daqu_A and Daqu_C compared to Daqu_B and Daqu_D, while dominant microorganisms showed the opposite pattern. Fungal community diversity was relatively constrained across all samples (Supplementary Figure 4). Previous studies indicate that rare taxa can enhance Baijiu fermentation efficiency (Mu et al., 2024), implying that Daqu_B and Daqu_D from the left riverbank may harbor unexplored functional microorganisms.

PCoA combined with PERMANOVA confirmed significant β-diversity differences among the four Daqu samples (*p* < 0.01), indicating substantial microbiota structural variations influenced by geographical distribution (Supplementary Figure 5). Daqu_B and Daqu_D exhibited greater diversity, with the river barrier can increasing community dissimilarity between banks.

**Figure 5:**
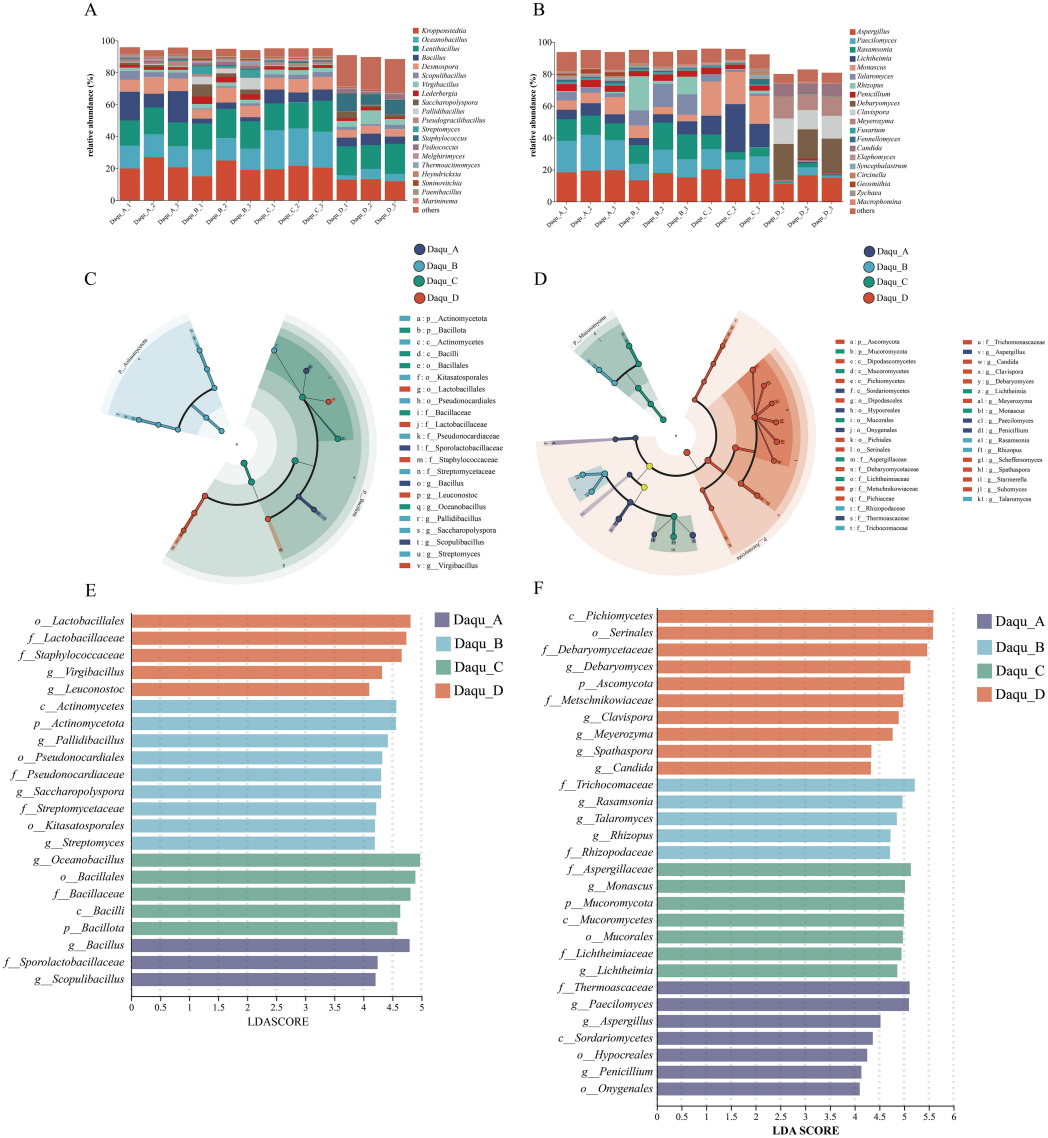
Microbial structure of high temperature Daqu. **(A)** bacteria and **(B)** fungi. Linear discriminant analysis effect size (LEfSe) of microbial communities amongst different high temperature Daqu (LDA score ≥ 4). **(C, E)** bacteria and **(D, F)** fungi.

Dominant bacterial genera in high-temperature Daqu included *Kroppenstedtia, Oceanobacillus, Lentibacillus, Bacillus, Desmospora, Scopulibacillus, Virgibacillus, Lederbergia, Saccharopolyspora*, and *Pallidibacillus*. Prominent fungal taxa included *Aspergillus, Paecilomyces, Rasamsonia, Lichtheimia, Monascus, Talaromyces, Rhizopus, Penicillium, Debaryomyces*, and *Clavispora* (Figures 5A, B), consistent with previous characterizations of Daqu microbiota sourced from Chishui River production area (Yang et al., 2024a,b). Significant differences in dominant microorganisms were observed between the Daqu samples from the two riverbanks, a finding that has not been reported previously.

On the left bank, *Kroppenstedtia* dominated bacterial communities (11.98%−24.90% from Daqu_D to Daqu_B), while *Oceanobacillus* decreased from 16.87 to 4.56% from Daqu_B to Daqu_D. Conversely, *Bacillus* and *Virgibacillus* proportions increased from Daqu_B to Daqu_D (3.19%−5.68% and 2.47%−5.38%, respectively). The fungal communities were dominated by *Aspergillus* (14.35%−15.56%), followed by *Paecilomyces* (2.23%−12.11%) and *Rasamsonia* (1.22%−14.75%).

On the right bank, several bacteria increased from Daqu_A to Daqu_C: *Oceanobacillus* (13.73%−23.47%), *Lentibacillus* (15.98%−17.46%), *Virgibacillus* (1.78%−2.83%), and *Pseudogracilibacillus* (1.90%−2.04%). Fungal communities showed increased abundance of *Lichtheimia* (6.48%−18.87%), *Monascus* (7.71%−19.74%), and *Fennellomyces* (1.24%−1.32%), while *Aspergillus* (19.19%−17.47%), *Paecilomyces* (20.30%−11.80%), and *Rasamsonia* (12.06%−6.43%) decreased.

LEfSe analysis revealed clear biomarker distinctions between the two riverbanks (Figures 5C–E). Right bank samples (Daqu_A and Daqu_C) were characterized by *Bacillus, Scopulibacillus, Aspergillus, Paecilomyces, Penicillium, Oceanobacillus, Lichtheimia*, and *Monascus*. Left bank samples (Daqu_B and Daqu_D) were defined by *Pallidibacillus, Saccharopolyspora, Streptomyces, Rasamsonia, Rhizopus, Talaromyces, Leuconostoc, Virgibacillus, Candida, Clavispora, Debaryomyces, Meyerozyma, Scheffersomyces, Spathaspora, Starmerella*, and *Suhomyces*. This striking divergence is consistent with a potential “ecological isolation” effect along the Chishui River, suggesting that the river may influence microbial dispersal patterns and contribute to the formation of distinct community profiles on each bank.

Consequently, the river functions as a natural biogeographical barrier that fundamentally shapes the microbial biodiversity of Daqu, ultimately contributing to regional variations in fermentation characteristics and product quality. Our findings align with prior studies identifying typical biomarker microorganisms in high-temperature Daqu, including *Oceanobacillus, Scopulibacillus, Bacillus, Streptomyces*, and *Rasamsonia* (Chen et al., 2025; Huang et al., 2024; Lei et al., 2025), further validating the consistency of microbial patterns across different Daqu studies.

*Bacillus* and *Oceanobacillus* contribute critically to Daqu's enzymatic potential: *Bacillus* synthesizes hydrolases, while *Oceanobacillus* produces α-amylase. These enzymes collectively catalyze the breakdown of macromolecules (e.g., starch and proteins) into glucose, amino acids, and various flavor precursors (Jiang et al., 2025; Zhu et al., 2025). Additionally, free amino acids generated by *Bacillus* enhance the baked aroma notes in Baijiu (Shen et al., 2020). Meanwhile, functional fungi such as *Rhizopus, Aspergillus, Monascus, and Candida e*xhibit high amylolytic activity, degrading starch into reducing sugars for yeast metabolism, or producing esterases that catalyze the formation of fruit-scented ester compounds (Wang et al., 2020; Xu et al., 2021, 2022).

**Figure 6:**
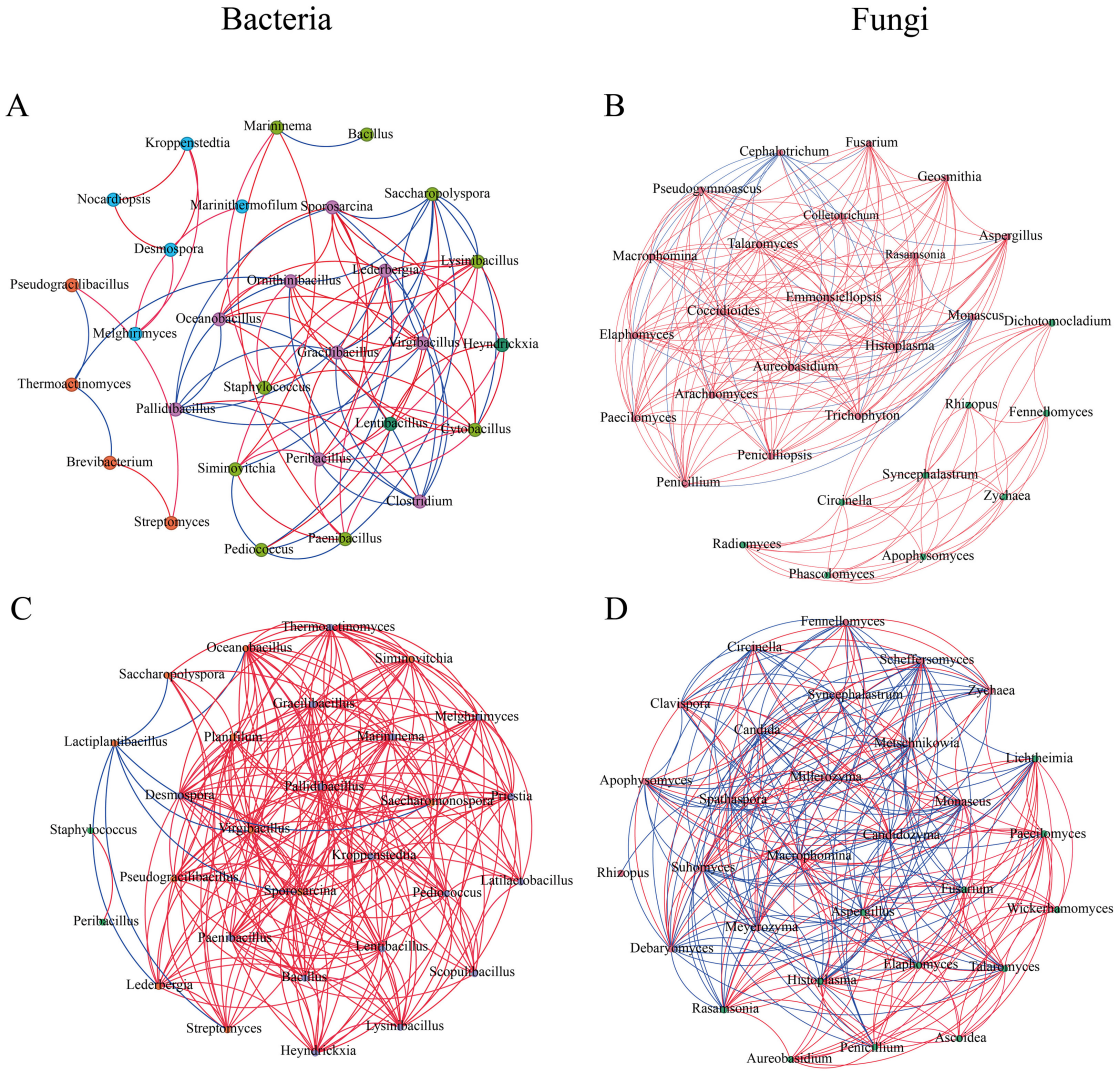
The co-occurrence networks in the right side of Chishui River **(A, B)**, left side of Chishui River **(C, D)**. The positive edges (Spearman's ρ > 0.6) are represented in red, and the negative edges (Spearman's ρ < −0.6) are represented in blue. (For interpretation of the references to color in this figure legend, the reader is referred to the web version of this article).

To elucidate microbial interactions, co-occurrence networks were constructed based on Spearman correlation analysis (|*r*| > 0.6, *p* < 0.05), including the top 30 most abundant genera (Figure 6). The enhanced bacterial cooperation observed on the left bank, characterized by higher average degree and positive correlations, signifies a synergistic metabolic state. This network topology suggests that bacterial taxa on the left bank are more likely to engage in cross-feeding and syntrophic interactions, which are crucial for the efficient production of enzymes and flavor precursors in the nutrient-rich HTD environment (Wu et al., 2025). Conversely, the reduced fungal interaction on the left bank suggests that environmental filtering may have promoted niche specialization among dominant molds, reducing the need for complex inter-taxa dependencies. These structural differences confirm that “ecological isolation” not only drives taxonomic divergence but also fundamentally reshapes the interaction patterns that underpin the fermentation process.

The bacterial network on the right bank of the Chishui River exhibited a notably higher proportion of negative correlations (34.48%) compared to the left bank (3.14%), approximately tenfold greater (Figures 6A, C). For fungi, the positive correlation coefficient was substantially higher on the right bank (86.47%) than on the left bank (54.25%) (Figures 6B, D).

Topological analysis further revealed structural differences: the bacterial network on the right bank had a density of 0.21 and an average degree of 6, whereas the fungal network showed higher connectivity, with a density of 0.50 and an average degree of 14.27. These values were lower than those on the left bank, where the bacterial network reached a density of 0.54 and an average degree of 15.37, and the fungal network attained a density of 0.64 and an average degree of 18.8. These results indicate that microbial communities on the left bank formed tighter ecological associations and were more conducive to microbial coexistence (Jiao et al., 2022).

Applying network topology measures such as closeness, centrality and degree to identify keystone taxa in the Daqu located on both sides of the Chishui River (Banerjee et al., 2018). Keystone taxa analysis revealed distinct microbial drivers on each riverbank. On the left bank of the Chishui River, keystone taxa included *Oceanobacillus, Virgibacillus, Sporosarcina, Macrophomina, Monascus*, and *Paecilomyces*. In contrast, right-bank communities were characterized by keystone taxa such as *Ornithinibacillus, Peribacillus, Thermoactinomyces, Penicillium, Elaphomyces*, and *Cephalotrichum*. This divergence in keystone profiles between riverbanks suggests that different microbial populations may potentially influence community assembly and succession processes on each side of the rive (Yan et al., 2025). Notably, although *Ornithinibacillus* and *Cephalotrichum* exhibited relatively low abundance (< 0.1%), they were identified as keystone taxa, highlighting their disproportionately important roles in shaping community dynamics and ecosystem development despite their low numerical representation (Riddley et al., 2025; Yang et al., 2024a).

The distinct network topologies on opposite banks underscore the “ecological isolation” driven by the Chishui River. Right-bank bacterial networks showed higher negative correlations, suggesting niche partitioning, while left-bank networks exhibited greater connectivity and cooperation. Crucially, low-abundance keystone taxa like *Ornithinibacillus* and *Cephalotrichum* serve as central “microbial hubs” despite their rarity (< 0.1%). We propose that the river barrier filters for these specific keystones, which then coordinate the metabolic flux of dominant species. For example, right-bank interactions likely promote the synthesis of pyrazine compounds, while left-bank architecture favors a different volatile profile. This link between network structure and flavor divergence explains how the geographical barrier ultimately shapes the regional identity of Daqu.

### 3.4 Functional differences of Daqu microbial communities

Four-dimensional label-free quantitative proteomics was employed to analyze the Daqu proteome. A total of 214,293 spectra were obtained, of which 212,099 were successfully matched, corresponding to 168,585 peptides, 35,610 identified proteins, and 35,229 quantified proteins.

**Figure 7:**
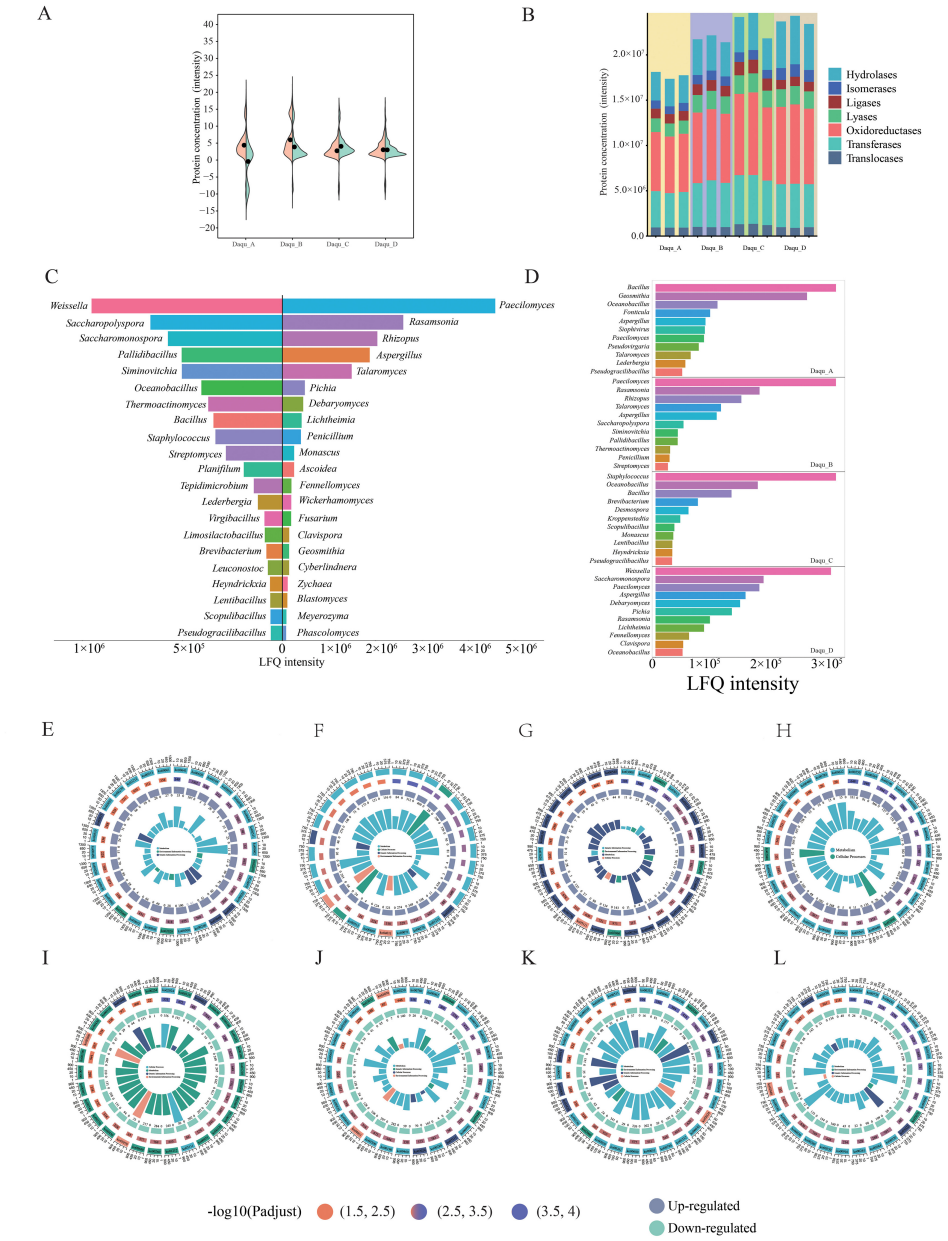
Functional microorganisms and their functional expression differences in different Daqu. Proteomic differences in microbial community expression of high temperature Daqu located on the both sides of the Chishui River **(A, B)**. Composition of functional microorganisms based on protein expression and dominant functional microorganisms (Relative abundance >1 %) **(C)** of Daqu located on the both sides of the Chishui River **(D)**. Functional differences of Daqu located on the both sides of the Chishui River. Enrichment circos metabolic pathways of significantly up-regulated (**E**: Daqu_A_vs_Daqu_C, **F**: Daqu_B_vs_Daqu_D, **G**: Daqu_A_vs_Daqu_B, **H**: Daqu_C_vs_Daqu_D), and down-regulated proteins (**I**: Daqu_A_vs_Daqu_C, **J**: Daqu_B_vs_Daqu_D, **K**: Daqu_A_vs_Daqu_B, **L**: Daqu_C_vs_Daqu_D) in Daqu.

Total microbial protein content was highest in Daqu _D, followed by Daqu _B, Daqu _C, and Daqu _A (*p* < 0.05; Figures 7A, B). In high-temperature Daqu, fungal communities were the predominant contributors of proteins, exhibiting 1.28-fold higher levels than bacterial proteins.

However, the activities of 1,712 enzymes did not strictly adhere to the overall trends described above. Specifically, hydrolases and isomerases showed the highest activity in Daqu_D, whereas transferases, lyases, ligases, and translocases were most abundant in Daqu_C. Moreover, Daqu_D exhibited markedly reduced enzyme levels compared to the other samples (Supplementary Figure 6). Examination of Daqu from different seasons revealed significant differences in protein expression (Yang S. et al., 2024; Yang et al., 2024b), this study also observed similar findings. Given the involvement of these enzymes in the metabolism of flavor-forming raw materials, a multi-omics approach is essential to comprehensively understand the functional differences among Daqu types.

The functional microorganisms in the four Daqu samples, collected from both sides of the Chishui River, were identified through protein expression analysis. A total of 32 microbial genera, defined as dominant based on a relative protein expression abundance greater than 1%, were identified. Among these, 14 genera including *Aspergillus, Bacillus, Oceanobacillus, Paecilomyces, Talaromyces, Rasamsonia, Penicillium, Streptomyces, Saccharopolyspora, Monascus, Pichia, Debaryomyces, Rhizopus*, and *Weissella* were shared across all samples (Figures 7C, D). These findings align with microbial community structure analyses. Key genera such as *Bacillus* and *Oceanobacillus* contribute markedly to the production of hydrolytic enzymes that break down macromolecules (Li et al., 2023), while *Monascus* and *Rhizopus* play essential roles in saccharification and flavor formation (Xu et al., 2022).

Although Daqu from different regions exhibit broadly similar functions, specific metabolic specializations were also observed (Figures 7E–L). For instance, compared to Daqu_B, Daqu_A showed up-regulation in arginine and proline metabolism, styrene degradation, and carbon fixation in photosynthetic organisms, but down-regulation of fatty acid biosynthesis, oxidative phosphorylation, and propanoate metabolism. Similarly, Daqu_C exhibited up-regulation of glycine, serine and threonine metabolism, aflatoxin biosynthesis, and the pentose phosphate pathway relative to Daqu_D, along with down-regulation of C5-branched dibasic acid metabolism, glycerophospholipid metabolism, and arginine biosynthesis.

When comparing Daqu from the same riverbank, Daqu_A up-regulated peptidoglycan biosynthesis, prokaryotic carbon fixation, and glyoxylate and dicarboxylate metabolism compared to Daqu_C, but down-regulated steroid biosynthesis and N-glycan biosynthesis. In contrast, Daqu_B up-regulated O-glycan biosynthesis, 2-oxocarboxylic acid metabolism, and mannose-type O-glycan biosynthesis relative to Daqu_D, while down-regulating the biosynthesis of plant secondary metabolites and alanine, aspartate, and glutamate metabolism.

Further analysis revealed that the “ecological isolation effect” of the Chishui River led to consistent functional changes in both cross-bank comparison groups (Daqu_A_vs._Daqu_B and Daqu_C vs. Daqu_D). Commonly upregulated functions included oxidative phosphorylation, starch and sucrose metabolism, glutathione metabolism, ether lipid metabolism, carbon fixation in photosynthetic organisms, styrene degradation, pentose phosphate pathway, and arginine and proline metabolism, as well as cysteine and methionine metabolism. Conversely, purine metabolism, monobactam biosynthesis, nitrogen metabolism, and pentose interconversions were consistently downregulated.

In comparisons between same-bank samples (Daqu_A vs. Daqu_C and Daqu_B vs. Daqu_D), coordinated transcriptional regulation was also observed. Functions commonly upregulated included valine, leucine, and isoleucine degradation; glycine, serine, and threonine metabolism; pentose phosphate pathway; fatty acid metabolism; cysteine and methionine metabolism; and selenocompound metabolism. Consistently downregulated functions were fatty acid biosynthesis, lysine degradation, monobactam biosynthesis, oxidative phosphorylation, starch and sucrose metabolism, and taurine and hypotaurine metabolism.

### 3.5 Assembly mechanism of the Daqu microbial community

Although community assembly processes, including both deterministic and stochastic mechanisms, are well-studied in plant and animal ecology, their role in food microbial ecosystems has been less explored (Louw et al., 2023). Understanding these processes is crucial for deciphering species coexistence and enabling targeted microbial regulation (Huang et al., 2024; Yang S. et al., 2024; Yang et al., 2024b).

Geographical distribution has emerged as a key factor influencing microbial community assembly (Ding et al., 2024; Guo et al., 2024; Jiao et al., 2022). Accordingly, we assessed the assembly processes in four geographically distinct Daqu samples using null models (Supplementary Figure 7).

Bacterial communities in upstream Daqu (both banks) were assembled primarily stochastically, whereas downstream samples underwent deterministic assembly. Regarding fungal communities, right-bank samples (Daqu_A and Daqu_C) exhibited stochastic assembly, while left-bank samples (Daqu_B and Daqu_D) showed deterministically assembled.

These divergent assembly patterns may be partially explained by the inherent physiological traits of the microbiota. Fungi in HTD, particularly dominant genera like *Aspergillus* and *Paecilomyces*, produce vast quantities of resilient spores that facilitate long-distance aerial dispersal across the river barrier. This high dispersal potential likely contributes to the stochastic assembly observed on the right bank. Conversely, the deterministic assembly prevalent on the left bank suggests that environmental filtering, driven by localized microclimatic conditions, exerts a stronger influence than dispersal, effectively “sorting” species based on their functional fitness regardless of their spore-forming capabilities.

These findings align with prior reports indicating that microbial assembly varies by region, for instance, stochasticity dominated in Deyang Daqu, while determinism prevailed in Luzhou and Chengdu samples (Ma et al., 2024). Together, the results underscore that not only temperature (Yang et al., 2024a,b), but also geographic location notably influence assembly processes, consistent with observed stratification patterns within individual Daqu blocks (Wen et al., 2024).

## 4 Conclusions

This study integrated GC-MS, LC-MS, and high-throughput phenotyping to compare the metabolic profiles, microbial composition, and functional features of four Daqu samples from opposite banks of the Chishui River. A total of 158 volatiles were identified, with significant differences in type and content between banks. Cross-bank comparisons (e.g., Daqu_A vs. Daqu_B) revealed a pronounced metabolic divergence, suggesting a reprogramming of core pathways such as nitrogen assimilation, central carbon metabolism, and cofactor biosynthesis, indicating potentially distinct functional architectures. In contrast, same-bank samples (e.g., A vs. C) showed more conserved core metabolism, differing mainly in specialized pathways like D-amino acid and flavor synthesis.

Microbial community analysis indicated enrichment of *Bacillus, Scopulibacillus, Aspergillus*, and *Monascus* in right-bank Daqu (A and C), whereas left-bank samples (B and D) were enriched with *Pallidibacillus, Saccharopolyspora, Streptomyces, Rhizopus*, and *Talaromyces*. Network analysis suggested stronger positive bacterial interactions but reduced fungal cooperation from the right to the left bank.

The “two mountains sandwiching one river” topography creates a confined canyon environment that may influence microbial dispersal, contributing to the observed “ecological isolation” effect.

This effect particularly influenced fungal community assembly, which shifted from stochastic processes on the right bank to deterministic assembly on the left. This effect on the functional expression of the microbial community in Daqu eventually led to the functional difference of Daqu on both sides of Chishui River and the function of Daqu was mainly expressed by fungi. These divergent enzymatic and metabolic profiles serve as a functional “blueprint” for the final spirit, providing essential precursors (e.g., pyrazines) for Baijiu flavor and establishing the scientific basis for micro-regional “terroir” and quality differentiation.

While this study highlights the impact of micro-geographical isolation, future research incorporating larger sample sizes, and cross-seasonal environmental variables is necessary to further validate these findings. Ultimately, our work provides a novel theoretical framework for precision brewing regulation and the protection of geographical indications (GI) in the traditional Baijiu industry.

## Data Availability

Due to confidentiality agreements, the data cannot be publicly deposited. However, the raw data supporting the findings of this manuscript will be made available by the authors to qualified researchers upon reasonable request.
