# Supplementary material for: Ecological isolation by the Chishui River drives microecology divergence in Daqu
Source: Front Microbiol. 2026 Mar 12;17:1786069. doi: 10.3389/fmicb.2026.1786069 (PMC13017894; doi:10.3389/fmicb.2026.1786069)
Supplement: Supplementary file 1 [file Supplementary_file_1.docx]

# **Supplementary Material for Ecological Isolation by the Chishui River Drives Microecology Divergence in Daqu**

**Dandan Song^1^, Xian Zhong^1^, Yaling Mu^2^, He Huang^3^, Liang Yang^1,*^**

^1^ School of Brewing Engineering, Moutai Institute, Renhuai 564501, China; songdd0330@163.com (D. S); zhongxian2025@163.com (X. Z.); moutaiyl0725@163.com (L. Y.)

^2^ Kweichow Moutai Co. Ltd., Renhuai 564501, China; Mu997270@163.com (Y.M.)

^3^ Department of Food Science & Technology, School of Agriculture & Biology, Shanghai Jiao Tong University, Shanghai 200240, China; huanghe0416@sjtu.edu.cn; (H. H.)

***The complete contact details of the corresponding author:**

Name: **Liang Yang**;

E-mail: moutaiyl0725@163.com;

Phone: +86-18786819825;

Mailing address: Department of Brewing Engineering, Moutai Institute, Renhuai 564507, China.


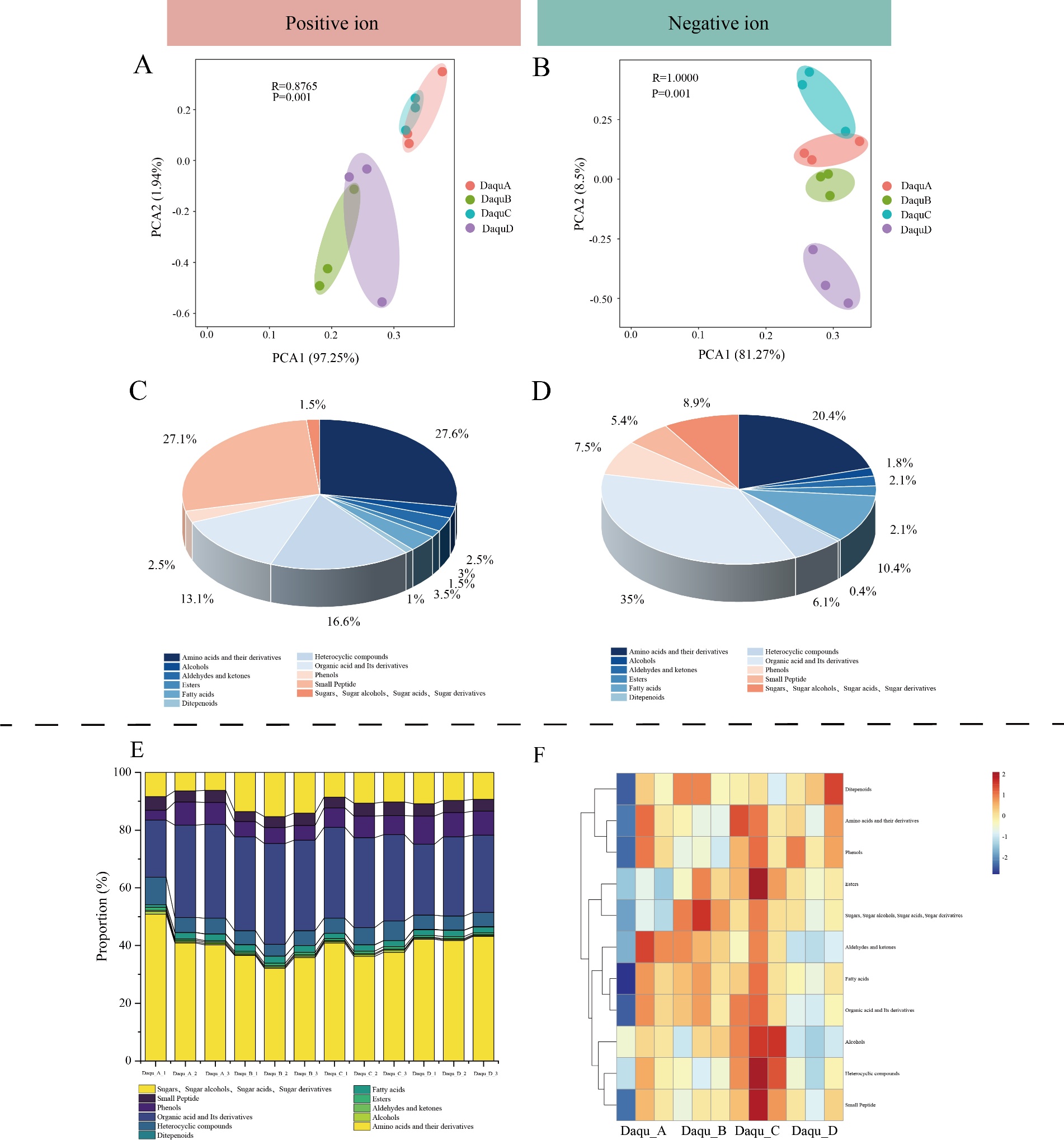


# **Fig. S1.** Overview of the distribution of non-volatile metabolites. (A-B) PCA analysis; (C-D) Classification of non-volatile metabolites; (E) Proportion of non-volatile compounds by category; (F) Variation in the total content of non-volatile compounds.


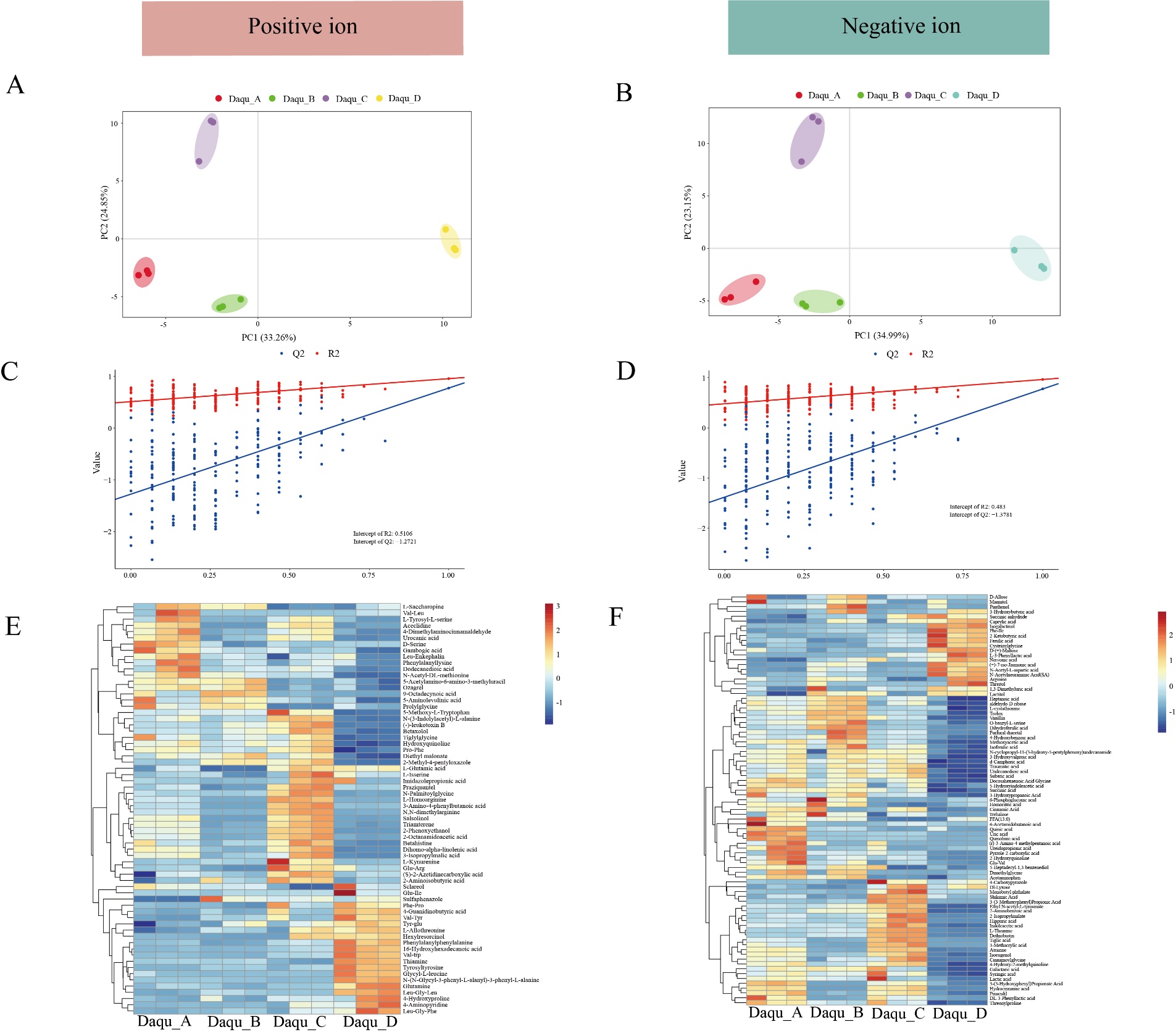


# Fig. S2. Distinction of different daqu by compound profiles. (A-D) PLS-DA plot of nonVOCs in soy sauce aroma and flavor type daqu. (E-F) Heatmap of differential microecological markers content in soy sauce aroma and flavor type daqu. Note: (For interpretation of the references to color in this figure legend, the reader is referred to the Web version of this article.)


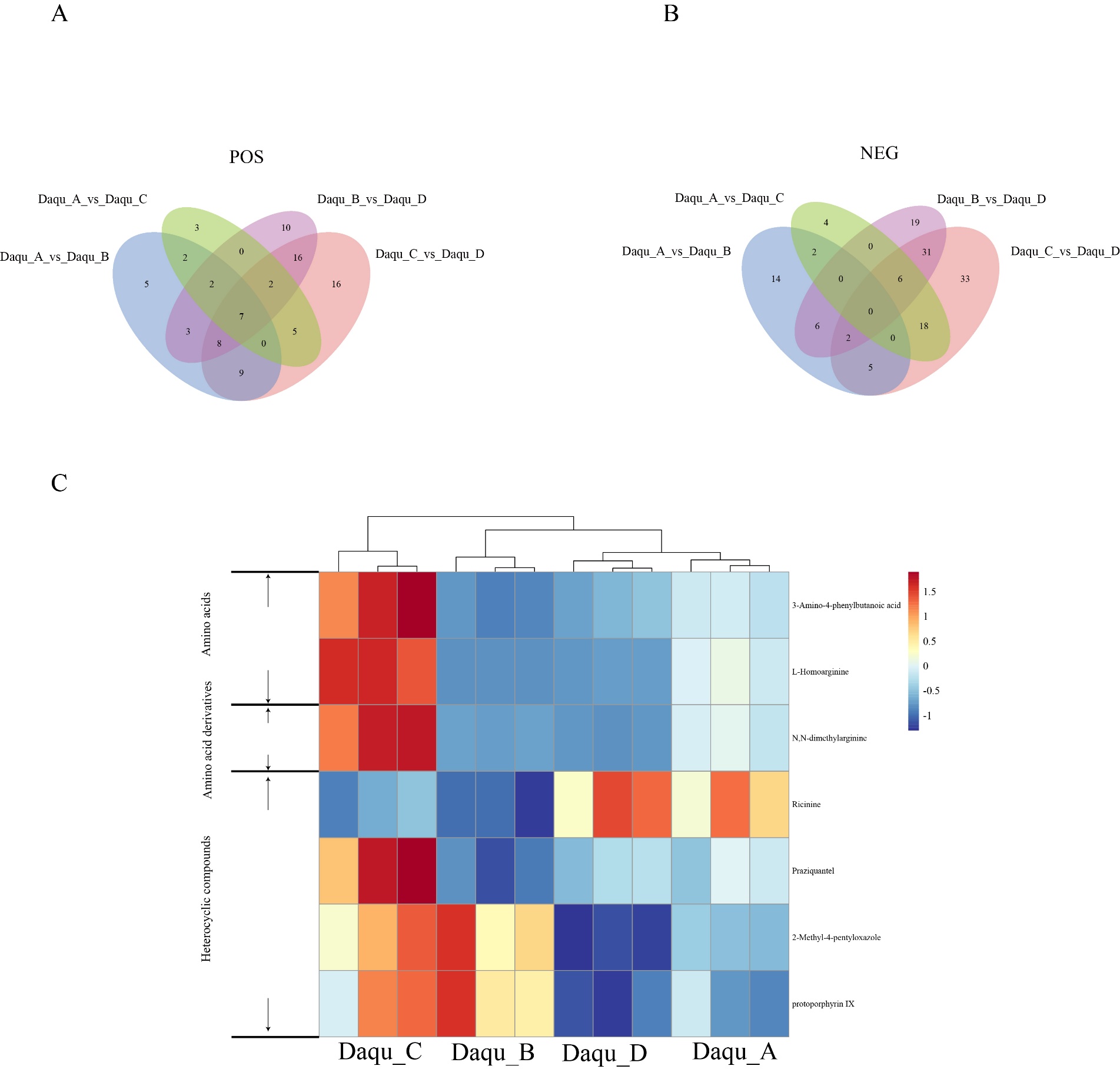


# Fig.S3. Venn analysis of differential metabolites between different Daqu Samples (A, B). Heatmap visualizes the composition of 7 non-volatile markers (C).


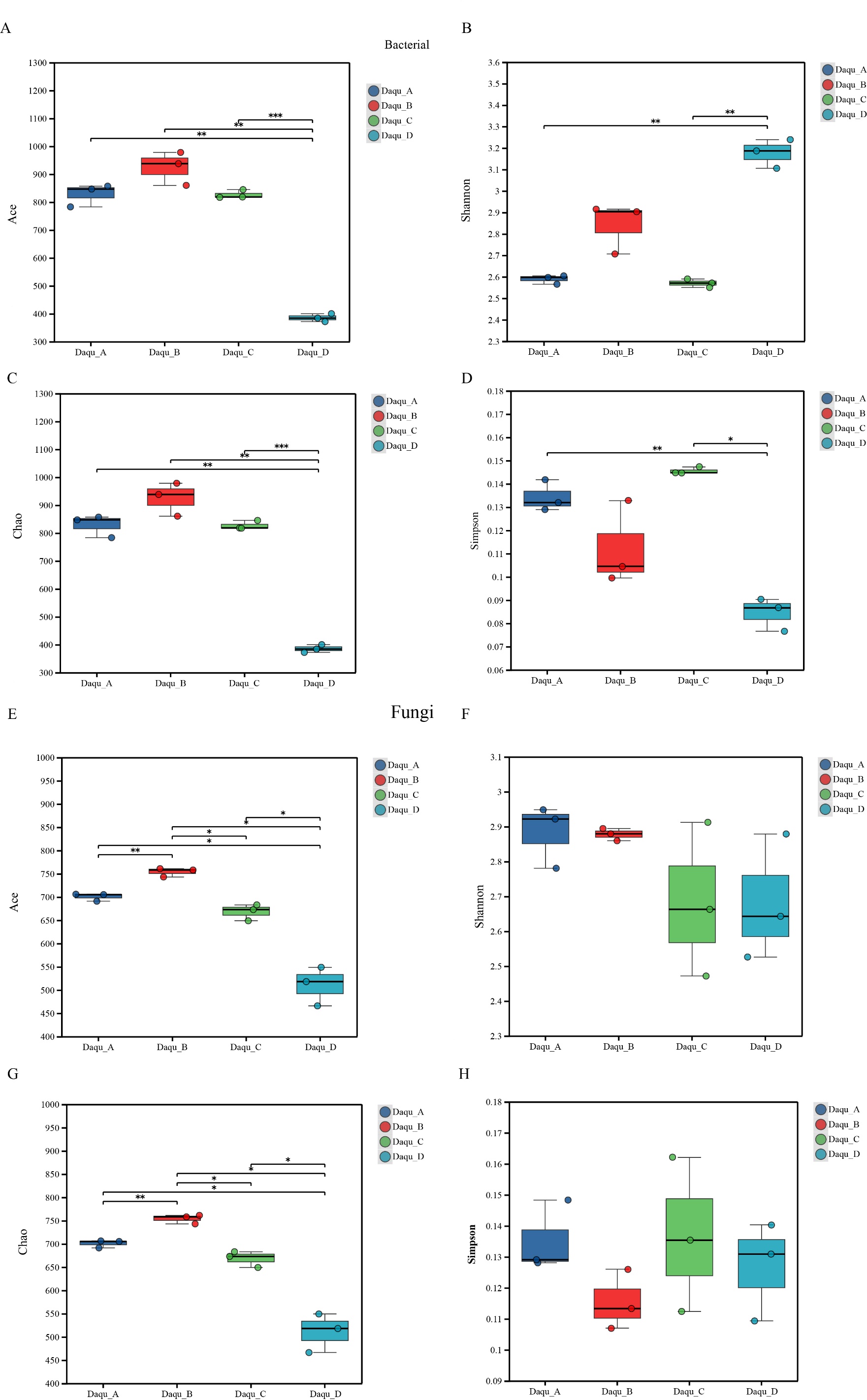


# Fig. S4 The α-diversities of microbial community within four Daqu. A-D:bacterial, (A) Ace. (B) Shannon. (C) Chao. (D) Simpson. E-H: fungi, (E) Ace. (F) Shannon. (G) Chao. (H) Simpson. *, *p* < 0.05; **, *p* < 0.01 for Kruskal-Wallis test.


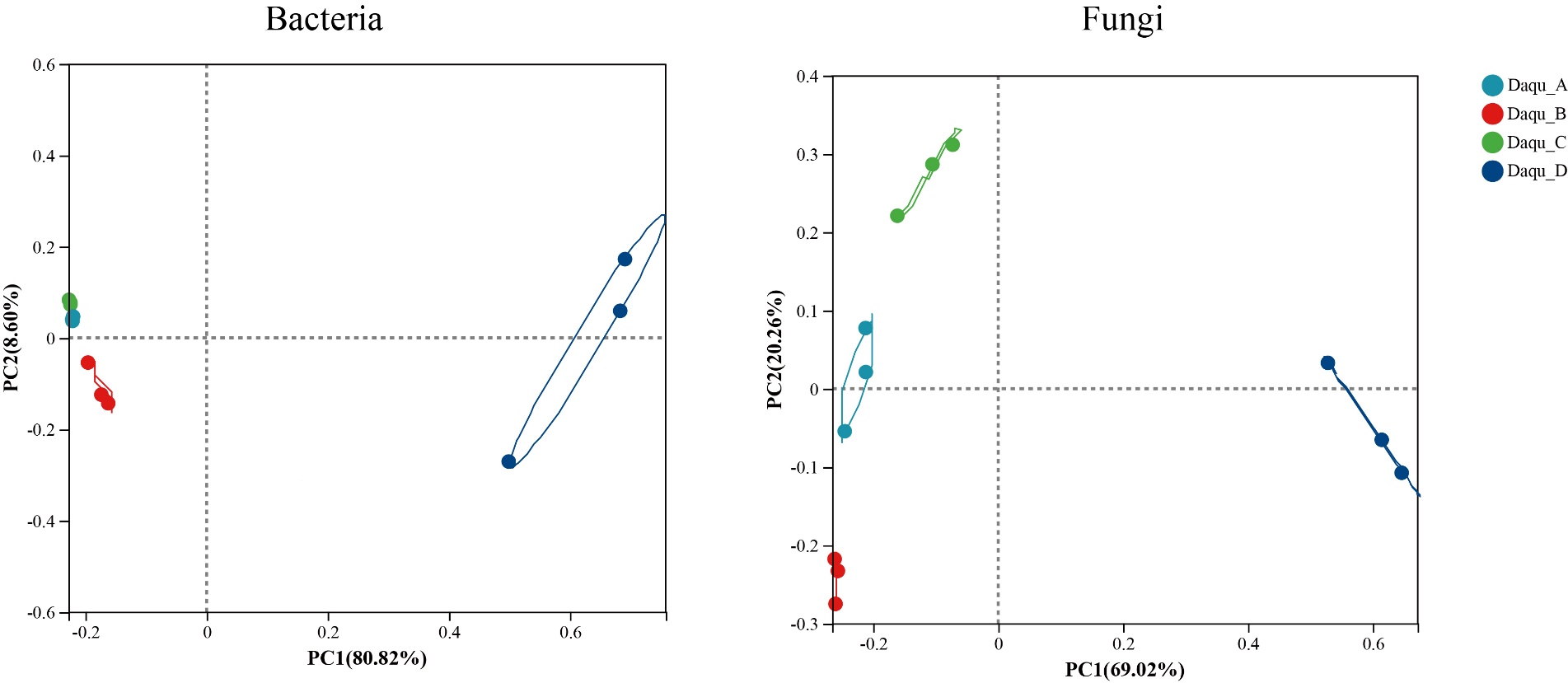


# Fig. S5. β-diversity index of Daqu microbial community. P<0.01.


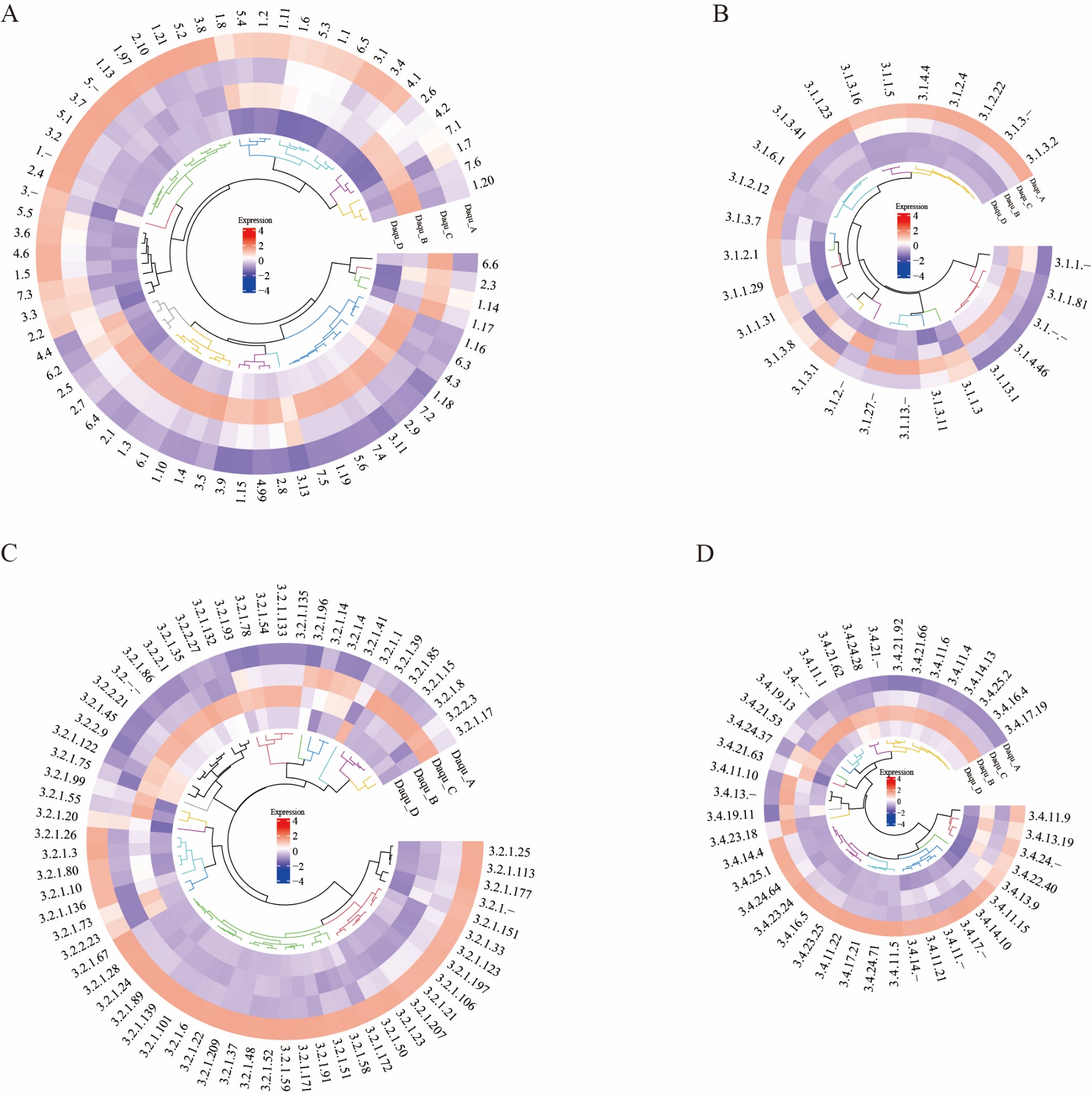


# Fig.S6. Expression of enzymes and functional microorganisms in Daqu after storage in different seasons. Differential expression of the seven major enzymes (A), hydrolytic enzymes such as esterase (B), glycosidase (C) and protease (D).


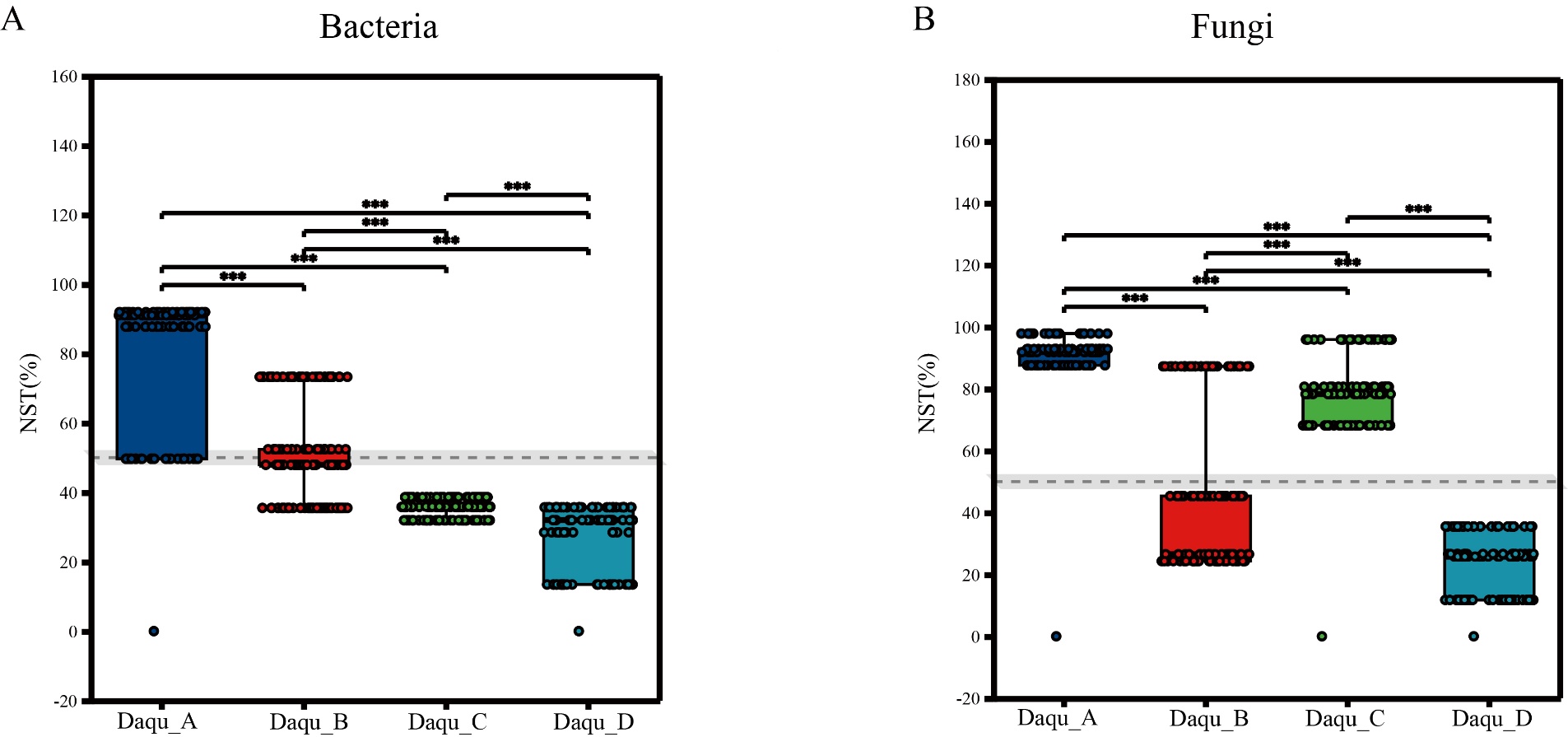


# Fig.S7. Ecological analysis showing the differences in betaNTI for bacterial (A) and fungal (B) communities of the four *Daqu*. NST<50%, NST>50% represent deterministic and stochastic processes, respectively.

# Tab.S1. Concentrations (μg/g) of volatile compounds in different types of daqu

|  | Daqu_A | Daqu_B | Daqu_C | Daqu_D |
| --- | --- | --- | --- | --- |
| Esters | 9.57±4.73 | 8.61±0.57 | 5.67±1.40 | 10.23±1.02 |
| Alcohols | 31.70±4.39b | 26.86±0.51b | 23.90±2.24b | 67.13±12.31a |
| Aldehydes and ketones | 14.13±1.48a | 19.71±2.57ab | 18.80±1.84ab | 22.62±3.99b |
| Fatty acids | 11.38±9.91 | 0.71±0.56 | 0 | 2.40±3.19 |
| Terpenes | 0b | 0.55±0.22a | 0b | 0b |
| Aromatic compounds | 8.04±0.78a | 15.39±0.36c | 9.91±1.23ab | 13.42±4.20bc |
| Phenols | 9.28±4.62 | 34.89±34.98 | 29.65±4.92 | 8.95±1.19 |
| Sulfur compounds | 0b | 0b | 3.85±0.33a | 0b |
| Furans | 1.22±0.49b | 1.59±0.25b | 0.30±0.22a | 1.60±0.07b |
| Pyrazines | 140.74±10.85a | 23.00±0.55b | 313.90±24.04c | 231.27±55.96d |
| Other nitrogen-containing and heterocyclic compounds | 1.47±0.14 | 0.71±0.24 | 2.03±1.22 | 2.21±0.52 |
| Lactones | 1.30±0.24b | 0a | 1.13±0.27b | 0a |
| Alkanes and alkenes | 1.49±0.32b | 6.43±2.94a | 1.71±0.41b | 1.48±0.33b |
| Others | 34.12±1.49b | 38.07±6.38b | 53.02±1.47a | 58.41±7.38a |

# Tab. S2. Statistics of metagenomic sequencing and bioinformatics analysis.

| Parameter | Daqu_A | Daqu_B | Daqu_C | Daqu_D |
| --- | --- | --- | --- | --- |
| Raw Data (bp) | 13, 328, 335, 422 | 12, 532, 787, 090 | 12, 665, 703, 028 | 14,465,093,622 |
| Target Reads Count | 87, 440,750 | 82, 998, 590 | 83, 878, 828 | 95, 795, 322 |
| Clean Q20 (%) | 99.28 | 99.27 | 99.32 | 99.35 |
| Clean Q30 (%) | 96.18 | 96.13 | 96.33 | 96.73666667 |
| High-quality Reads (%) | 98.96 | 98.84 | 98.97 | 99.41 |
| Contigs Number | 107805 | 195824 | 122982 | 58521 |
| N50 Length (bp) | 4476 | 1921 | 2096 | 1406 |
| ORFs Number | 236244 | 384539 | 252743 | 90817 |
| Unigenes Number | 133576 | 185667 | 131956 | 51549 |
